# Sesn2 attenuates the damage of endothelial progenitor cells induced by angiotensin II through regulating the Keap1/Nrf2 signal pathway

**DOI:** 10.18632/aging.104156

**Published:** 2020-11-24

**Authors:** Shiao Ding, Nan Ma, Hao Liu, Min Tang, Ju Mei

**Affiliations:** 1 Department of Cardiothoracic Surgery, Xinhua Hospital, School of Medicine, Shanghai Jiao Tong University, Shanghai, China

**Keywords:** atherosclerosis, endothelial progenitor cells, angiotensins, sestrin 2, Nrf2

## Abstract

Endothelial progenitor cell (EPC) dysfunction is an important physiopathological mechanism in the dynamics of the formation of atherosclerosis. It has been reported that angiotensin II (Ang-II) damages the function of EPCs in atherosclerotic plaque through induction of oxidative stress. Sestrin 2 (Sesn2) serves as an antioxidant role in oxidative stress, however, the exact mechanisms underlying the dynamics of how Sesn2 may factor into EPCs after Ang-II treatments needs to be illustrated. We isolated EPCs from human umbilical cord blood samples and treated with Ang-II. Western blotting, qRT-PCR, transwell assays, immunofluorescence and so on were used to investigate the mechanisms underlying the roles of Sesn2 in EPCs treated with Ang-II. Ang-II was found to promote the apoptosis of EPCs as well as inhibited the mRNA and protein expression of Sesn2. Upregulation of Sesn2 attenuated the negative effect of Ang-II. Sesn2 increased the protein expression of Nrf2 by enhancing P62-dependent autophagy. Silencing of Nrf2 enhanced the degree of apoptosis of EPCs as well as resulted in the impairment of EPC functions through inducing the promotion of (reactive oxygen species) ROS production. Our study results indicated that Sesn2 facilitated the viability of EPCs After treatment with Ang-II, as well as provided a potential therapeutic target to alleviate the progression of atherosclerosis.

## INTRODUCTION

Arterial hypertension is still a global health problem, significantly leading to cardiovascular related mortality [1]. The low effectiveness of currently available therapies for arterial hypertension results in a high risk of developing atherosclerosis, cerebral stroke, and acute coronary events. Internal bleeding from microvessels related with vasa vasorum contributes to the instability and rupturing of atherosclerotic plaque in arterial hypertension [2]. These microvessels originate from endothelial progenitor cells (EPCs). Hypertension can cause EPC function impairments, which lead to the reduction of EPCs’ angiogenesis function. First, in hypertension patients, decline in EPC function seems to be more common and appears to occur earlier than the decline in the number of EPCs [3–6]. Second, Antihypertensive treatment can reduce EPC count and restore EPC function [7, 8]. Third, Different types of EPCs may by affected differently in hypertension patients, and the proliferation activity of late EPCs is significantly lower than that of other types of EPCs [9, 10]. In the process of angiogenesis, through the proliferation and migration of endothelial cells, the initial vascular plexus is remodeled and refined to form new blood vessels [11]. In this process, ECs are tightly combined to form the lumen of blood vessels [12]. Many factors may damage endothelial cells, including physical damage, biochemical damage and immune-mediated damage. When this happens, endothelial cells cannot maintain homeostasis, which is called endothelial dysfunction, which may lead to cardiovascular disease such as atherosclerosis. In patients with coronary heart disease, as the severity of the disease increases, the number of EPCs and circulating EPCs gradually decreases. EPCs contribute to postpartum vascular repair and neovascularization. The EPC function of patients with hypertension and diabetes is impaired, inhibiting the endogenous repair of vascular disease, leading to the progression of atherosclerosis [13]. Ang-II has been detected at high concentrations in atherosclerotic plaques and hypertension patients and plays a key role in inducing resultant damage in functions of, as well as promotes the apoptosis of EPCs [14–17].

EPCs were first discovered by Asahara in 1997, are a group of precursor cells which can circulate, proliferate, and differentiate into endothelial cells [18]. Emerging evidence has demonstrated that EPCs circulating in the blood can induce significantly impairment with their functions in the dynamics of endothelium regulation and protection [3, 19]. However, cardiovascular risk factors could also impair the viability and function of circulating EPCs [3, 20, 21]. Many studies have shown that oxidative stress may be one of the most important and major factors in such types of dynamics, and can induce resulting in dysfunctional and numerically decreased EPCs in cases of cardiovascular disease [20, 22, 23]. Restraint of the production of reactive oxygen species (ROS) has been reported to concomitantly result in the reduction of the abundances and functions of EPCs [23]. However, the mechanisms underlying the impairment of EPCs in the hypertension are mostly unknown.

Sestrin 2 (Sesn2) is a member of a family of proteins including Sestrin 1, 2, and 3 [24]. These forms of Sestrin are conserved stress-responsive proteins recently first given formal names during a research conference on human genetics [25]. Sestrin proteins have been identified to be able to protect cells from oxidative stress induced by low and decreasing levels of H_2_O_2_. Depletion of Sesn2 in cultured cells has been found to cause the accumulation of ROS [26]. On the other hand, conditionally induced overexpression of Sesn2 has been found to prevent cells from undergoing early deaths caused by many factors such as hypoxia, glucose deprivation, and H_2_O_2_, which is suggestive of the idea that Sesn2 possesses antioxidant functions [27]. Nuclear factor erythroid 2-related factor 2 (Nrf2) is a famous transcription factor that improves activities of antioxidant enzymes and which increases the expression of many cytoprotective genes in response to oxidative or other stresses [28–30]. Cross talk between Sesn2 and Nrf2 protects cell metabolism in the dynamics of antioxidant activities [31]. Sesn2 has been found to promote autophagic-dependent degradation of Kelch-like ECH-associated protein 1 (Keap1) and thereby activating Nrf2 signaling pathway which leads to the increased levels of activity of antioxidant genes [32, 33]. In some research findings, Nrf2 is postulated to play antioxidant functions dependent upon the downstream functions of Sesn2 [34]. Additional research findings have indicated that in the process of autophagic flux, the adaptor protein P62 (also known as sequestosome 1) directly promotes Sesn2-dependent activation of Nrf2 [33]. Research by Lao et al. [35] indicated that Sesn2 promoted HUVEC survival after the treatment with Ang-II. Their research also indicated that Ang-II improved the levels of Sesn2 through inducing activation of the JNK/c-Jun pathway in HUVEC and thus indicated that Sesn2 protects endothelial cells. However, their research was only focused upon the upstream mechanisms of Sesn2 in HUVEC while the downstream mechanisms of Sesn2 in the dynamics of endothelial cells remains as unknown.

Therefore, we would postulate that that Sesn2 could attenuate the Ang-II-induced apoptosis of EPCs by way of upregulating the expression of Nrf2 protein. Sesn2 caused the increased expression of Nrf2 through enhancing the flux of autophagy. Thus, our study was designed to analyze whether Sesn2 facilitate the survival and function of EPCs via promoting Nrf2 expression after treatments with Ang-II. Taken together, we hoped that our efforts would increase the understanding of mechanisms underlying the influences of Sesn2 in the dynamics of EPCs as well as provide a potential therapeutic method for treatment of hypertension.

## RESULTS

### EPCs identification and detection of Sesn2 and Nrf2 protein expression in EPCs treated with Ang-II

**Figure 1:**
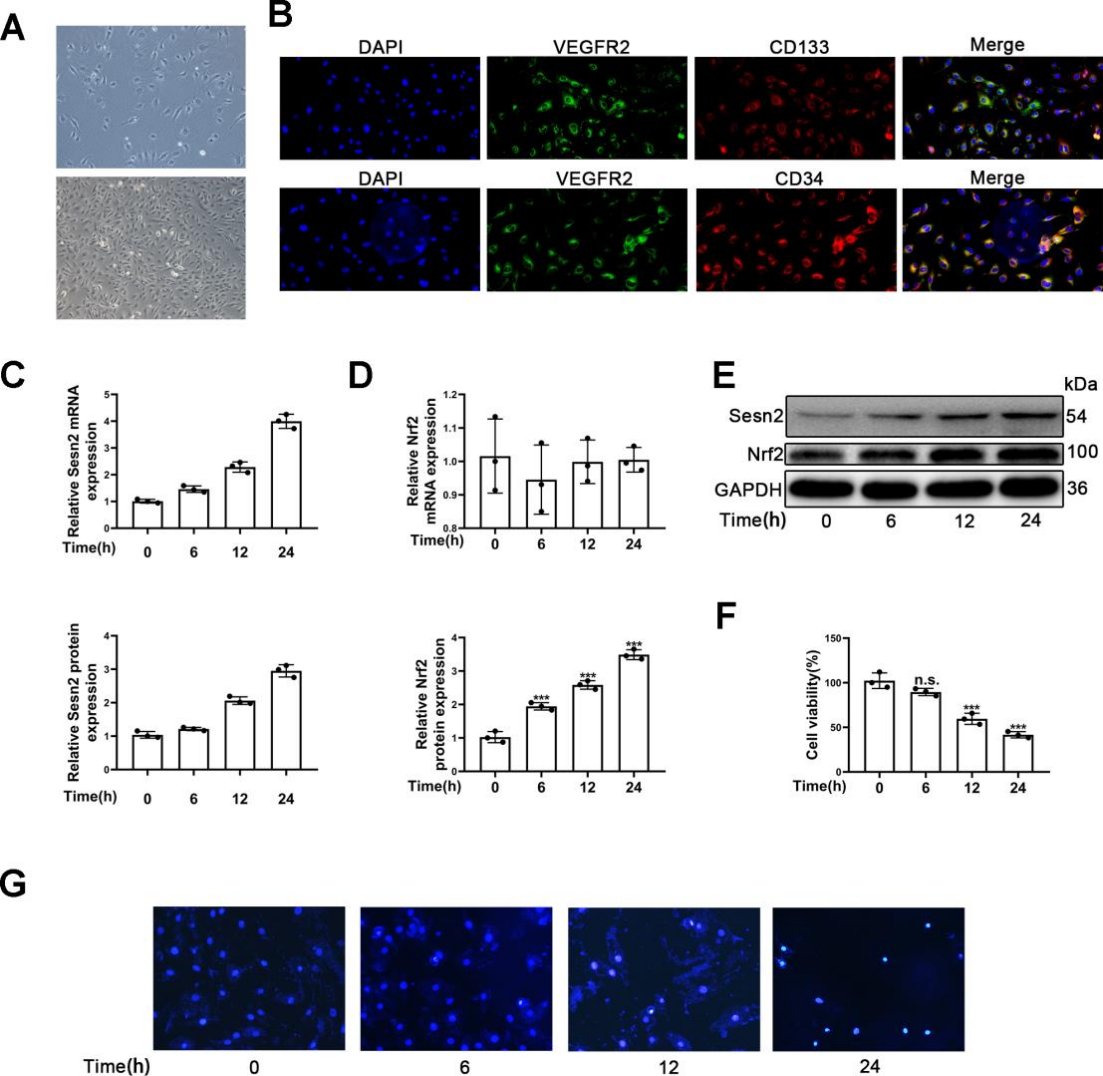
**Identification of EPCs and Ang-II increased Sesn2 and Nrf2 protein expression in EPCs.** Identification and exposures of EPCs to Ang-II for prolonged periods of time. (**A**) EPCs (passage (P) 1) from human umbilical cord. Scale bars = 5 μm and Scale bars = 40 μm. (**B**) We used immunofluorescence to detect the measures of CD31, CD34, CD134, and VEGFR2. (**C**) The treatments with Ang-II caused an increased levels of Sesn2 mRNA in EPCs. (**D**) The levels of Nrf2 mRNA were unchanged in EPCs exposed to Ang-II. (**E**) The treatments with Ang-II increased the levels of Sesn2 and Nrf2 proteins in EPCs. (**F**) We used Hoechst 33258 to detect the apoptosis of EPCs and found that the treatments with Ang-II caused an increase in the apoptosis of EPCs. Scale bars = 5 μm. All experiments were performed in triplicate. *p < 0.05, **p < 0.01, ***p < 0.001, versus the control. Data are represented as mean +/- SEM. (**G**) We used Hoechst 33258 staining to detect the apoptosis of EPCs. Scale bars = 5 μm.

Firstly, we observed that the morphology of isolated cells exhibited a cobblestone pattern, which is considered to be typical morphology of EPCs (Figure 1A). Secondly, results indicated that the isolated cells expressed CD31, CD34, CD133, and VEGFR2, all of which are thought to be typical markers of EPCs (Figure 1B). Flow cytometry results showed that the positive rate of CD34, CD133 and VEGFR2 was 75.9%±1.6, 48%±2.1 and 81.9%±1.9% respectively (Supplementary Figure 1). Further, it is accepted that VEGFR2 is a classical endothelial cell marker and accepted that D34 and CD133 are classical progenitor cell markers. Therefore, the results suggested that EPCs expressing CD31, VEGFR2, CD34, and CD133 were late-EPCs.

To investigate whether or not the expression of Sesn2 and Nrf2 in EPCs treated with Ang-II compared to untreated samples, we treated EPCs with 1 μM Ang-II over predetermined times (i.e., 0, 6, 12, 24 h). We used a dose of Ang-II according to information available in previous research [36–38]. The mRNA levels of Sesn2 and Nrf2 were detected by using qRT-PCR. The results indicated that Sesn2 mRNA expression was enhanced for a prolonged time (Figure 1C). In contrast the results suggested that Nrf2 mRNA expression had not changed in EPCs over the prolonged time (Figure 1D). Moreover, our results from Western blotting assessments revealed that the treatment of Ang-II induced increases in the protein levels of Sesn2 and Nrf2 (Figure 1E). The levels of Nrf2 proteins in nuclear of EPCs were also increased (Supplementary Figure 2D). These results showed that the entry of Nrf2 to nuclear of EPCs was enhanced significantly. Ang-II also caused the increased levels of LC-3 II and caused the decreased levels of P62 (Supplementary Figure 2A). The levels of autophagy were found to have been promoted by the treatment with Ang-II. In order to examine the role of Ang-II application in EPCs, we used Hoechst33258 to determine the apoptosis of EPCs, and we used CCK8 to detect the cell viability of EPCs. These results indicated that the apoptosis was increased and the cell viability was reduced in EPCs exposed to Ang-II for prolonged time (Figure 1F, 1G). These results indicated that autophagy, Sesn2, and Nrf2 might be involved in the dynamics of apoptosis of EPCs induced by the treatment of Ang-II. Further, there may be complex mechanisms and interactions between autophagy, Sesn2, and Nrf2 in EPCs exposed to Ang-II.

### Sesn2 attenuates the apoptosis of EPCs induced by Ang-II through promoting the protein level of Nrf2

**Figure 2:**
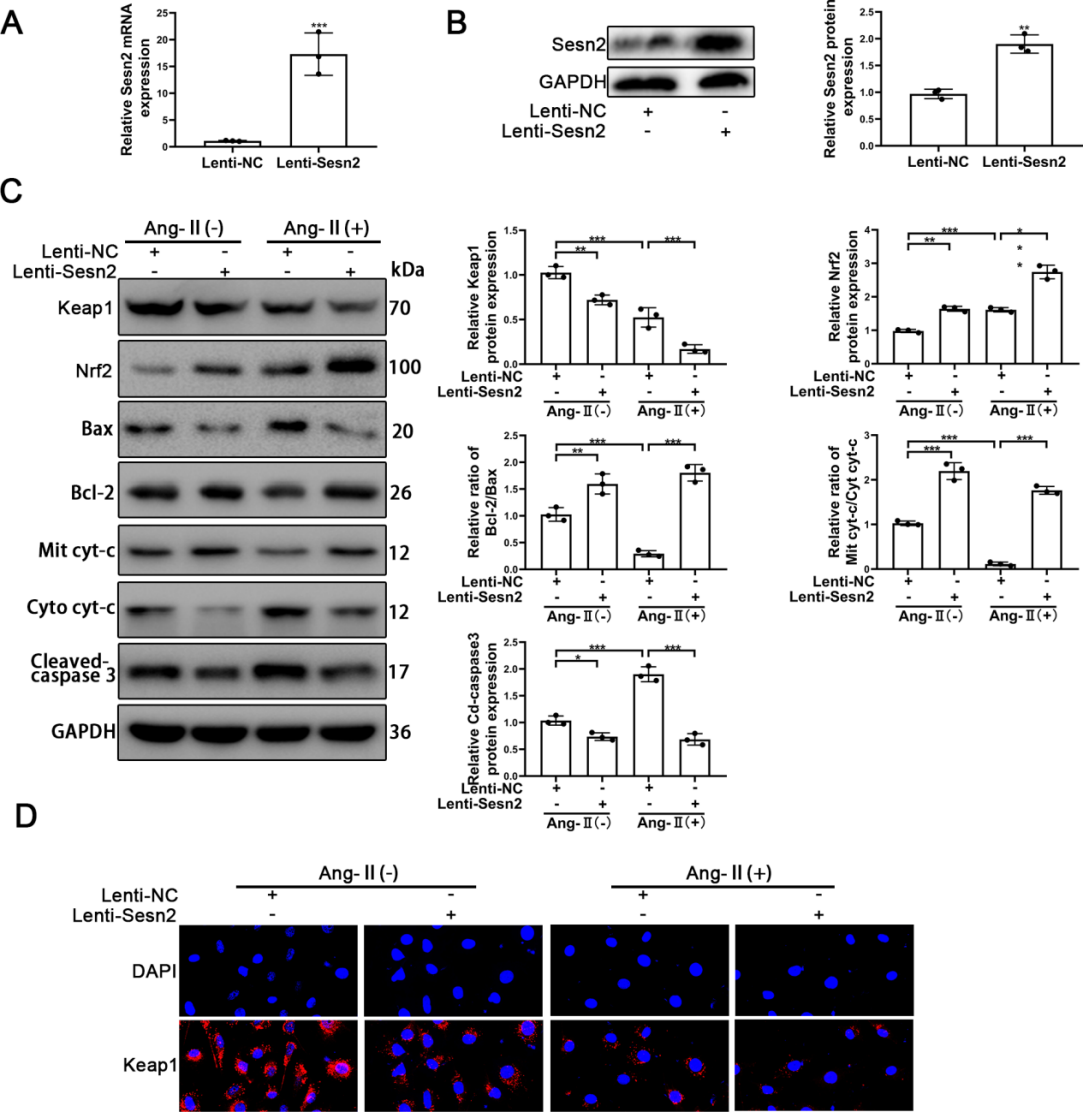
**Overexpression of Sesn2 promoted the survival of EPCs after Ang-II treatment.** EPCs were transfected with Lenti-Sesn2 or NC before the treatments with Ang-II. (**A**) The effects of Lenti-Sesn2 were confirmed by using qRT-PCR. (**B**) The levels of Sesn2 proteins were upregulated significantly by the applications of Lenti-Sesn2. (**C**) The levels of Keap1, Nrf2, Bax, Bcl-2, Mit cyt-c, Cyto cyt-c, Cleaved-caspase 3 and GAPDH related proteins in EPCs were detected by using Western blotting. (**D**) The levels of Keap1 proteins in EPCs were detected by using Immunofluorescence. Scale bars = 2 μm. All experiments were performed in triplicate. *p < 0.05, **p < 0.01, ***p < 0.001, versus the control. Mit cyt-c: Mitochondria cytochrome C; Cyto cyt-c: Cytoplasm cytochrome C. Data are represented as mean +/- SEM.

To investigate the role of Sesn2 in the EPCs treated with Ang-II, the LV3-pGLV-H1 + Puro plasmids with pcDNA-Sesn2 or control oligonucleotides (Lenti-Sesn2 and Lenti-NC) were designed such as to overexpress Sesn2 protein. The effect of inducing overexpression of Lenti-Sesn2 was confirmed by using qRT-PCR and Western blotting. The levels of Sesn2 mRNA were found to have been remarkably upregulated, and the levels of Sesn2 proteins were also significantly overexpressed (Figure 2A, 2B). We established that we achieved stable transfection of Lenti-Sesn2 and Lenti-NC in EPCs and these EPCs were used in following experiments. Recent research has reported that Keap1 induced downregulation of the levels and activity of Nrf2 [29, 30]. Therefore, the levels of Nrf2 and Keap1 proteins were measured by using Western blotting. As could be seen in Figure 2C, the treatments with Ang-II induced the decreased levels of Keap1 proteins and contrastingly increased the levels of Nrf2 proteins and the entry of Nrf2 to nuclear in EPCs (Supplementary Figure 2E). Interestingly, upregulation of Sesn2 was found to have further reduced the levels of Keap1 proteins and the entry of Nrf2 to nuclear in EPCs (Supplementary Figure 2E). We used immunofluorescence in order to further detect the levels of Keap1 proteins. The results indicated that levels of Keap1 proteins were reduced by the treatments of Ang-II. The immunofluorescence results indicated that there was downregulation of Keap1 proteins after the treatments of Ang-II, which Sesn2 upregulation consequently was able to further enhance this effect (Figure 2D). These results suggested that the levels of Nrf2 proteins were negatively regulated by Keap1 in EPCs. Thus, we postulated that upregulation of Sesn2 induced repression of Keap1 and thereby enhanced the levels of Nrf2 proteins.

The role of Sesn2 in EPCs treated with Ang-II needed to be confirmed as part of our study and research goals. The treatments with Ang-II were found to have induced apoptosis of EPCs. The treatments with Ang-II promoted the flow of cyt-c from mitochondria to cytoplasm, changed the protein levels of Cleaved-caspase3, and reduced the relative ratio of Bcl-2/Bax (Figure 2C). The promotion of these proteins meant that apoptosis was induced by Ang-II treatments in EPCs. An important finding was that overexpression of Sesn2 could alleviate apoptosis of EPCs induced by the treatments of Ang-II (Figure 2C). Besides, Sesn2 upregulation could enhanced the tube formation of EPCs. Importantly, Sesn2 upregulation also could repair the impaired tube formation of EPCs induced by Ang-II treatments (Supplementary Figure 3A). In sum, these results indicated that Sesn2 could be used to help protect EPCs from apoptosis induced by Ang-II.

### Knockdown of Nrf2 can reverse the protective effect of Sesn2 on EPCs

**Figure 3:**
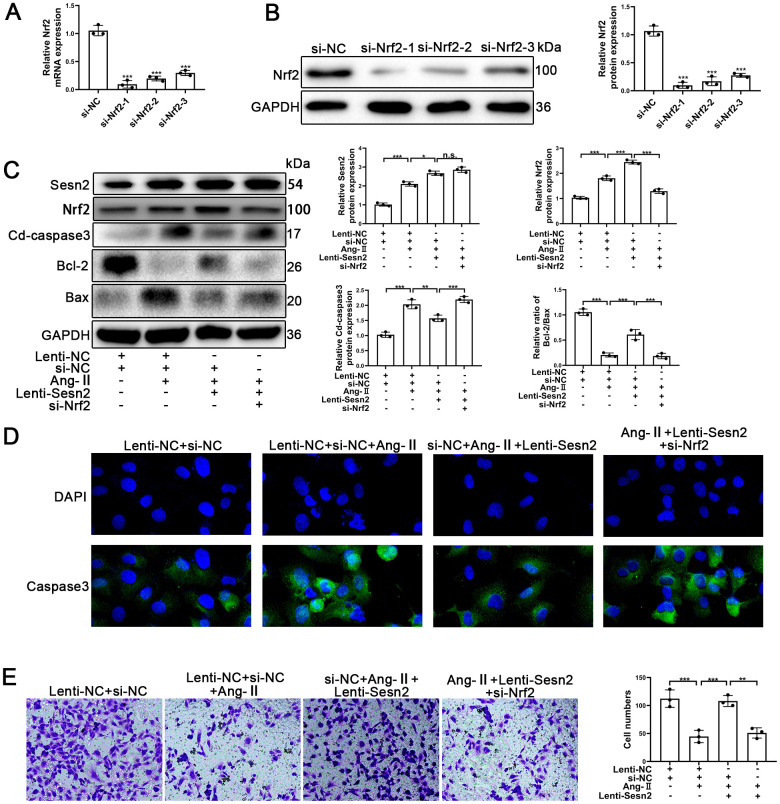
**Knockdown of Nrf2 could reverse the protective effect of Sesn2 in EPCs treated with Ang-II.** EPCs were divided into treatment groups including for a control, Ang-II, Ang-II + Lenti-Sesn2, and Ang-II+ Lenti-Sesn2 + si-Nrf2 treatment groups. (**A**) The levels of Nrf2 mRNA were found to have decreased in EPCs transfected with siNrf2. (**B**) The levels of Nrf2 proteins were found to have been reduced in EPCs transfected with si-Nrf2. (**C**) Measures for Sesn2, Nrf2, Cleaved-caspase3, Bcl-2, Bax, and GAPDH protein expression in EPCs were determined by using Western blotting. (**D**) We used immunofluorescence to detect levels of caspase3 protein in ECPs. Scale bars = 2 μm. (**E**) We determined the migration of EPCs by using Transwell assays. Scale bars = 5 μm. All experiments were performed in triplicate. *p < 0.05, **p < 0.01, ***p < 0.001, versus the control. Data are represented as mean +/- SEM.

To further investigate whether or not Sesn2 protected EPCs from Ang-II treatment through promotion of Nrf2, we designed Small interfering RNA (si-RNA) to silence the expression of Nrf2 mRNA and proteins. EPCs containing Lenti-Sesn2 or Lenti-NC were transfected with si-Nrf2. As could be seen in Figure 3A, 3B, the levels of Nrf2 mRNA were reduced significantly, and the levels of proteins also were decreased remarkably in EPCs. We used Si-Nrf2-1 in the following experiments described hereafter. Firstly, we used Western blotting to detect the apoptotic markers including such as Cleaved-caspase3, Bcl-2, and Bax. Our results indicated that upregulation of Sesn2 induced decreased levels of Cleaved-caspase3 but silencing of Nrf2 was able to induce reversal of this role in EPCs (Figure 3C and Supplementary Figure 1D). There were opposite effects with regards to the ratio of Bcl-2/Bax in EPCs (Figure 3C and Supplementary Figure 2F, 2D). Importantly, we observed that silencing of Nrf2 did not result in a reduction of the levels of Sesn2 in Lent-Sesn2 + Ang-II + si-Nrf2 treatment group. Our results also indicated that upregulation of Sesn2 could enhance the levels of Nrf2 and the entry of Nrf2 to nuclear in the Lent-Sesn2 + Ang-II + si-NC treatment group (Figure 3C and Supplementary Figure 2F). These results suggested that Sesn2 promoted the levels of Nrf2 proteins. The results from immunofluorescence assessments confirmed that although the levels of Cleaved-caspase 3were downregulated by Lenti-Sesn2, in contrast, si-Nrf2 enhanced the levels of expression of Cleaved-caspase3 in EPCs (Figure 3D). In order to verify whether or not si-Nrf2 had an influence upon the migration of EPCs, we used transwell assays. The results from these assessments indicated that the migration of EPCs were repressed by the treatments of Ang-II and indicated that Sesn2 could reverse this effect. However, silencing of Nrf2 resulted in the inhibition of the positive effect of Sesn2 on EPCs exposed to Ang-II (Figure 3E). These results revealed that Sesn2 protected EPCs from Ang-II through promoting the expression of Nrf2.

### Autophagy acts as an important mediator in Sesn2/Nrf2 sighing pathway

**Figure 4:**
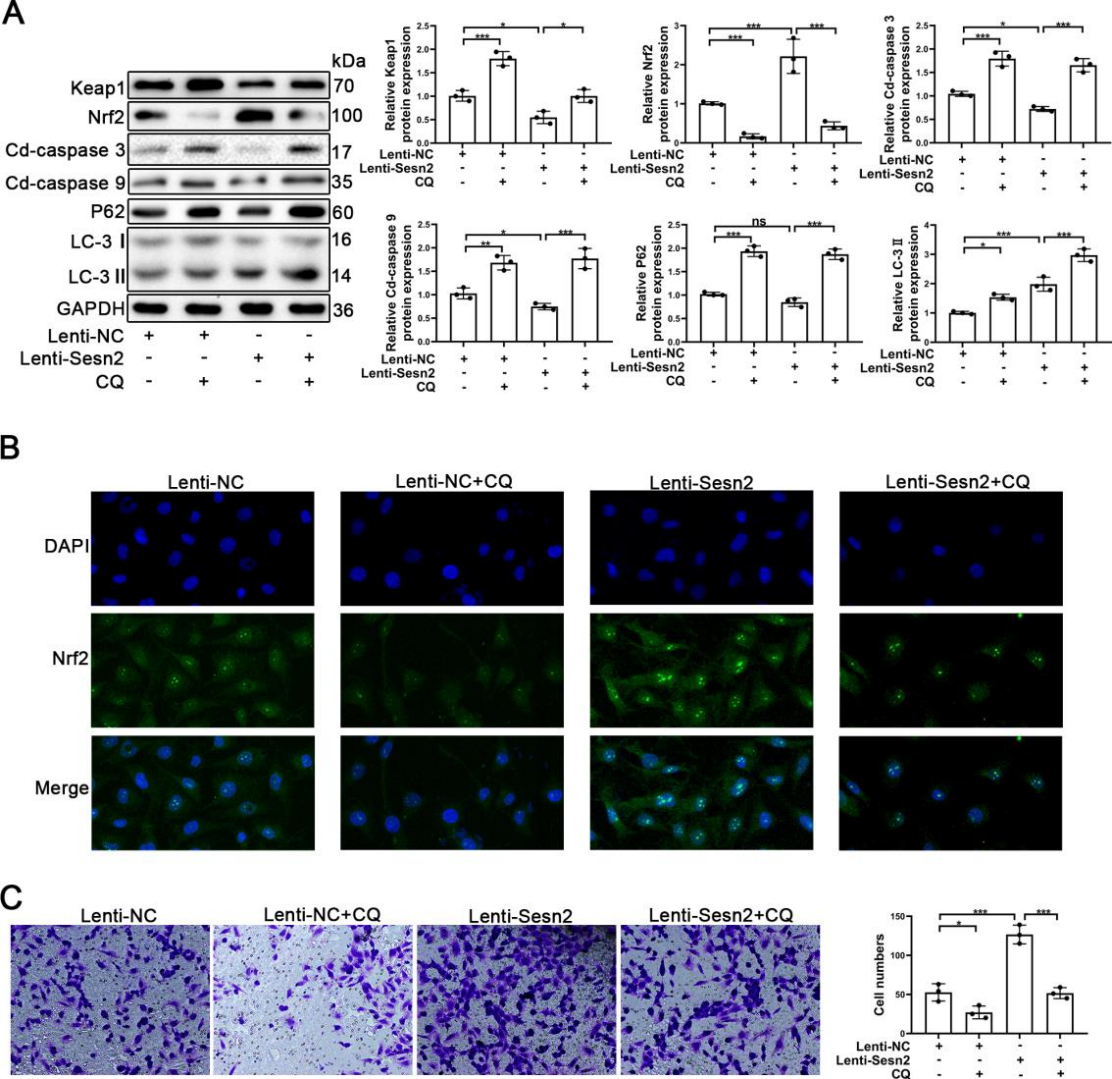
**Repression of autophagy inhibited the protective effect of Sesn2 in EPCs.** EPCs were divided into treatment groups representing the control, CQ, Lenti-Sesn2, and Lenti-Sesn2 + CQ cohorts. (**A**) The levels of expression of proteins of Keap1, Nrf2, Cleaved-caspase3, Cleaved-caspase9, P62, LC-3, and GAPDH in EPCs were detected by use of Western blotting. (**B**) The levels of expression of proteins of Nrf2 in EPCs were determined by using Immunofluorescence. Scale bars = 2 μm. (**C**) Measures of the migration of EPCs were determined by using Transwell assays. All experiments were performed in triplicate. Scale bars = 5 μm. *p < 0.05, **p < 0.01, ***p < 0.001, versus the control. Data are represented as mean +/- SEM.

An important aspect of our research was to determine how Sesn2 promotes Nrf2 in EPCs as previously this was unknown. Previously, autophagy has been reported to serve as a mediator between Sesn2 and Nrf2. In the process of autophagy, Sesn2 was found to have induced the degradation of Keap1 and thereby promoted Nrf2 signaling through P62-dependent autophagy [33, 39]. To confirm the role of autophagy between Sesn2 and Nrf2, we used chloroquine (CQ, 10 μM) to treat EPCs transfected with either Lenti-Sesn2 or Lenti-NC. Our results indicated that CQ treatments alone were able to repress the levels of autophagy flux and influenced the dynamics of the Keap1/Nrf2 pathway. Restraint of autophagy was capable of inducing the impaired viability of EPCs (Figure 4A) [40, 41]. Accordingly, our results indicated that upregulation of Sesn2 was able to induce autophagy. Treatment with CQ further inhibited autophagy that was induced by overexpression of Sesn2 (Figure 4A). Chloroquine acted as an inhibitor of lysosomes and inhibited the fusion of autophagosomes and lysosomes. In this manner, the levels of LC-3II were increased as the autophagic flux was inhibited. The levels of expression of P62 confirmed such findings (Figure 4A). Interestingly, our results indicated that Keap1 was negatively expressed in EPCs when Sesn2 was correspondingly overexpressed. And, then consequently, Nrf2 protein expression and the entry to nuclear was increased in EPCs (Figure 4A and Supplementary Figure 3B). However, our results indicated that CQ inhibited autophagic flux induced by Sesn2, and then inhibited the degradation of Keap1. Further, Keap1 repressed the levels of Nrf2. Meanwhile, Keap1 combined with Nrf2and thereby resulting in the retention of Nrf2 in cytoplasm (Figure 4A and Supplementary Figure 3B). We used immunofluorescence to determine the distribution of Nrf2 in EPCs. The results clearly indicated that Sesn2 promoted Nrf2 to enter the nucleus of EPCs while CQ reversed this phenomenon (Figure 4B). Finally, we evaluated the effect of autophagy upon the migration of EPCs and found that CQ could reduce the promotion of migration of EPCs by Sesn2. These results demonstrated that autophagy might have acted as an important mediator in the Sesn2/Nrf2 signaling pathway.

**Figure 5:**
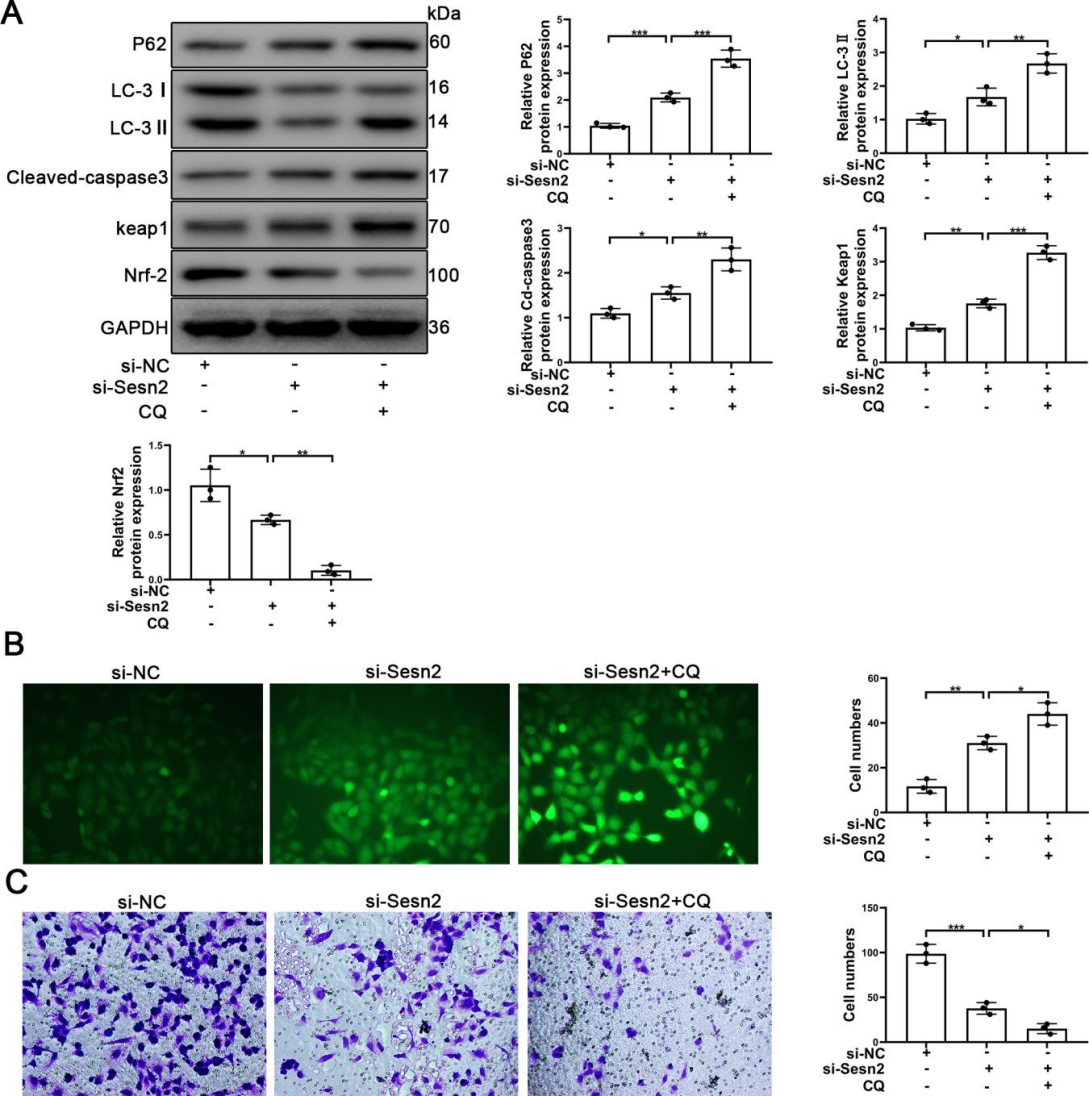
**Sesn2 protected the EPCs through regulating the autophagic flux.** EPCs were divided into treatment groups representing the control, si-Sesn2, and si-Sesn2 + CQ. (**A**) The levels of P62, LC-3, Cleaved-caspase3, Keap1, Nrf-2, and of GAPDH proteins in EPCs. (**B**) Measures of ROS production in EPCs were assessed by the use of fluorescent dye H2DCFDA. Scale bars = 5 μm. (**C**) Measures of the migration of EPCs were determined by using Transwell assays. Scale bars = 20 μm. All experiments were performed in triplicate. Scale bars = 5 μm. *p < 0.05, **p < 0.01, ***p < 0.001, versus the control. Data are represented as mean +/- SEM.

To further determine whether or not autophagy mediated the Sesn2/Nrf2 signaling pathway, we designed si-RNA to silence the expression of Sesn2. As shown in Supplementary Figure 2B, 2C, the levels of Sesn2 mRNA and proteins were reduced dramatically. We used si-Sesn2-1 in the following experiments described hereafter. Silencing of Sesn2 was found to have inhibited the upstream dynamics of autophagic flux, whereby we observed a decrease in LC-3 and an increase in P62 (Figure 5A). Meanwhile, the levels of Keap1 proteins were enhanced whereas the levels of Nrf2 and the entry of Nrf2 to nuclear were reduced significantly (Figure 5A and Supplementary Figure 3C). The rates of EPCs apoptosis also were found to have been increased when Sesn2 was silenced by si-Sesn2 (Figure 5A). Interestingly, CQ was found to have inhibited the fusion of autophagosomes and lysosomes, which ultimately resulted in the inhibition of the downstream dynamics of autophagy. Thus, the levels of P62 and LC-3 were increased when EPCs were treated with CQ (Figure 5A). The CQ treatment was found to have further suppressed Nrf2 levels and the entry to nuclear because the CQ treatment reduced the degradation of Keap1 through P62-dependent autophagic flux (Figure 5A and Supplementary Figure 3C). Many studies have already reported that as an antioxidant transcription factor, Nrf2 can activate many antioxidant enzymes such as to repress ROS levels in cells [42–44]. In our experiments, we found that ROS levels were increased remarkably in EPCs transfected with si-Sesn2 and found that CQ enhanced this effect (Figure 5B). Moreover, silencing of Sesn2 was found to have also inhibited the migration of EPCs, and CQ further repressed the migration of EPCs (Figure 5C). In summary, these results indicated that Sesn2 upregulated the Keap1/Nrf2 signaling pathway through inducing autophagy.

### Upregulation of Nrf2 promotes the viability and tube formation ability of EPCs

**Figure 6:**
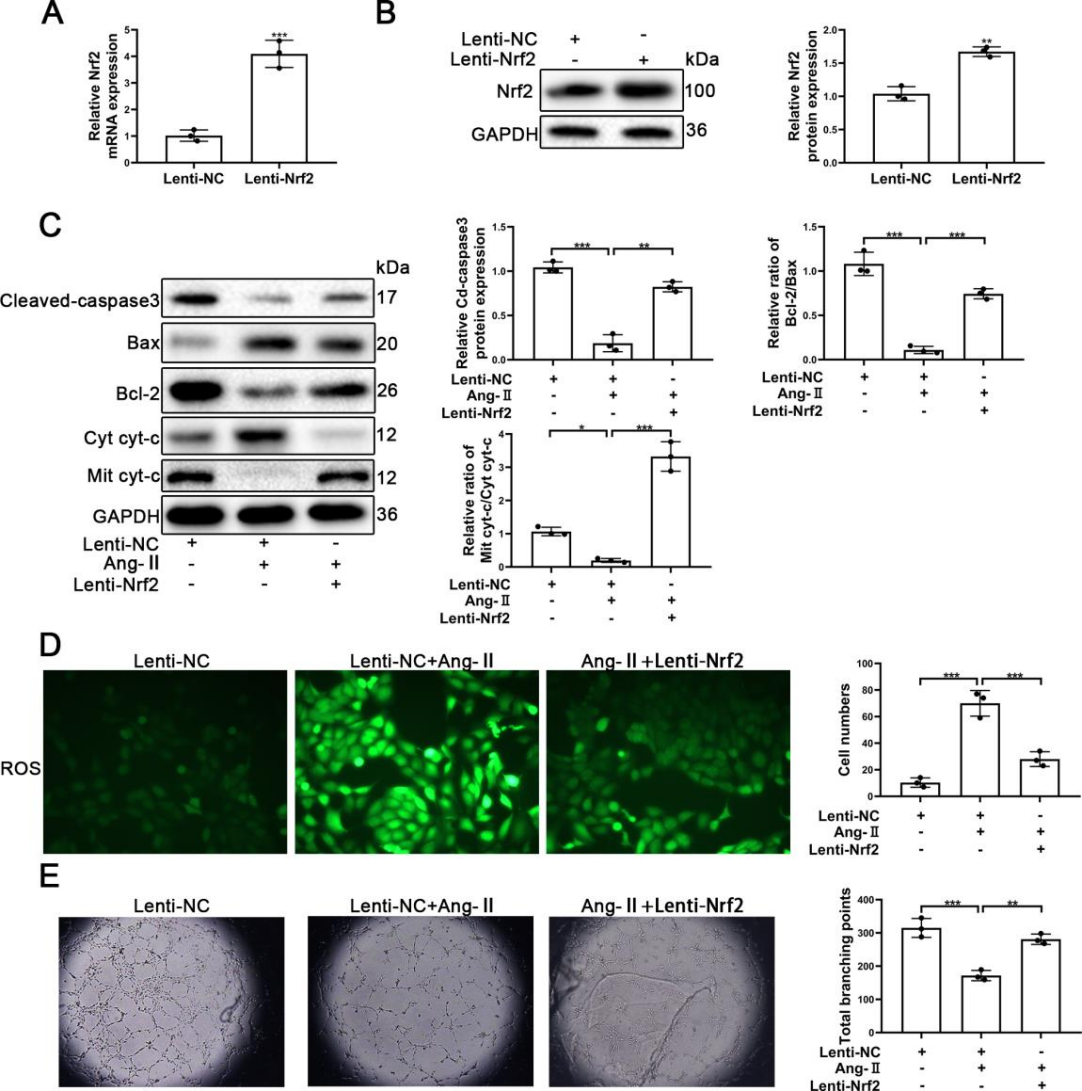
**Upregulation of Nrf2 inhibited Ang-II-induced apoptosis and dysfunction of EPCs.** EPCs were divided into treatment groups representative of the control, Ang-II, and Ang-II + Lenti-Nrf2. (**A**) The levels of Nrf2 mRNA were found to have increased significantly in EPCs transfected with Lenti-Nrf2. (**B**) The levels of Nrf2 proteins were found to have increased significantly in the Nrf2 samples transfected with Lenti-Nrf2. (**C**) The levels of for Bax, Bcl-2, Mit cyt-c, Cyto cyt-c, Cleaved-caspase 3, and GAPDH proteins in EPCs were detected by using Western blotting. (**D**) ROS production in EPCs was detected by the use of fluorescent dye H2DCFDA. Scale bars = 5 μm. (**E**) Tube formation abilities were assessed on Matrigel. All experiments were performed in triplicate. Scale bars = 5 μm. *p < 0.05, **p < 0.01, ***p < 0.001, versus the control. Data are represented as mean +/- SEM.

To confirm the role of Nrf2 in Ang-II-treated EPCs, the LV3-pGLV-H1 + Puro plasmids with pcDNA-Nrf2 or control oligonucleotides (Lenti-Nrf2 and Lenti-NC) were designed to overexpress levels of Nrf2 proteins. As shown in Figure 6A, the levels of Nrf2 mRNA were significantly increased. Nrf2 protein expression also was enhanced significantly by the treatment of Lenti-Nrf2 (Figure 6B). Our previous results indicated that Ang-II treatments was able to facilitate apoptosis of EPCs. In this section, our approach was to have transfected EPCs with Lenti-Nrf2 or Lenti-NC before the treatments with Ang-II. Overexpression of Nrf2 was able to induce the alleviation of apoptosis of EPCs that had been caused by the treatments of Ang-II. Nrf2 was found to have reduced the leaking of cyt-c from mitochondria to the cytoplasm. Nrf2 also caused a decrease of Cleaved-caspsae3 and an increase of Bcl-2/Bax (Figure 6C). Nrf2 usually serves as an antioxidant transcription factor which helps to inhibit ROS production in cells. Therefore, we detected the levels of ROS by use of the fluorescent dye H2DCFDA. Our results indicated that Nrf2 could reduce the levels of ROS caused by the Ang-II treatments. We found that green staining positive cells were reduced significantly in the Ang-II + Lenti-Nrf2 treatment groups (Figure 6D). It is understood that tube formation and migration abilities reflect the viability of EPCs. Thus, in order to investigate whether or not Nrf2 promoted tube formation and the migration ability of EPCs treated with Ang-II, we performed Matrigel angiogenesis assays and transwell assays. Our results indicated that Ang-II repressed the tube formation of EPCs and indicated that upregulation of Nrf2 could reverse this effect (Figure 6E). The results of transwell assays indicated that Ang-II inhibited the migration of EPCs and indicated that overexpression of Nrf2 could reverse this effect (Supplementary Figure 3D). These results suggested that upregulation of Nrf2 attenuated Ang-II-induced inhibition of the viability and the tube formation ability in EPCs.

### Nrf2 protects EPCs from Ang-II through inhibiting ROS production

**Figure 7:**
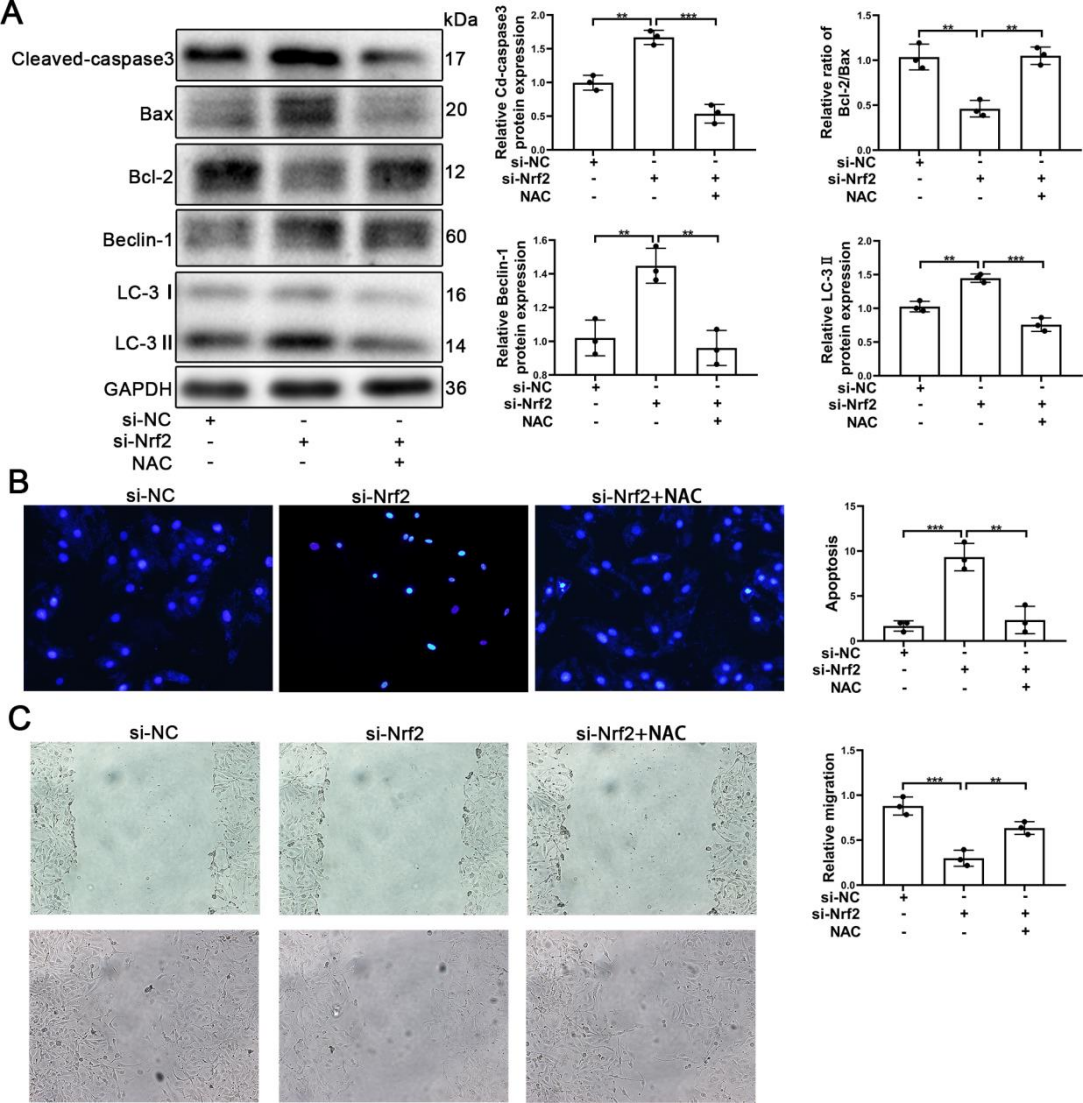
**NAC facilitated the survival and migration of EPCs after silencing of Nrf2.** EPCs were divided into treatment groups representative of the control, si-Nrf2 and, si-Nrf2 + NAC. (**A**) The levels of Cleaved-caspase3, Bax, Bcl-2, Beclin-1, LC-3, and GAPDH proteins in EPCs were determined by using Western blotting. (**B**) We used Hoechst 33258 staining to detect the apoptosis of EPCs. Scale bars = 5 μm. (**C**) Migration of EPCs was determined by the application of wound healing assays (details given in methods). All experiments were performed in triplicate. Scale bars = 20 μm. *p < 0.05, **p < 0.01, ***p < 0.001, versus the control. Data are represented as mean +/- SEM.

It is known that Nrf2 plays an important role in oxidative stress and that Nrf2 protects tissues and cells from oxidative stress. The results of our analyses using Western blotting indicated that silencing of Nrf2 was able to increase the autophagy and apoptosis. Further, knocking-down of Nrf2 caused an increase of Cleaved-caspse3 and caused a decrease of the Bcl-2/Bax ratios (Figure 7A). Similarly, knocking-down of Nrf2 induced an increase in the levels of expression of Beclin-1 and LC-3 (Figure 7A). Thus, we postulated that promotion of autophagy might have played a protective role in EPCs when Nrf2 was silenced.

NAC (N-acetyl-L-cysteine, 10mM) [45] was used to investigate whether or not Nrf2 protected EPCs by suppressing ROS production. EPCs transfected with si-Nrf2 were treated with NAC. As can be seen in Figure 7A, Cleaved-caspase3 was found to have decreased and Bcl-2/Bax was found to have increased, indicating that apoptosis of EPCs induced by silencing of Nrf2 was reduced by NAC. Our results also indicated that autophagy was decreased in EPCs transfected with si-Nrf2. The results of Hoechst33258 confirmed that NAC attenuated the apoptosis of EPCs (Figure 7B). Importantly, our results indicated that NAC was capable of facilitating the migration and the tube formation ability of EPCs transfected with si-Nrf2 (Figure 7C and Supplementary Figure 3E). These results demonstrated that Nrf2 protected EPCs through inhibiting ROS production.

## DISCUSSION

Firstly, as has been found in previous research, our own study confirmed the that Ang-II could induce the apoptosis and dysfunction of EPCs in the process of hypertension [25, 46–48]. We also found that Ang-II promoted the expression of Sesn2 and Nrf2 proteins in EPCs. The levels of mRNA Sesn2 were also enhanced by the treatments of Ang-II. However, the levels of Nrf2 mRNA remained unchanged after we applied Ang-II treatments. These results suggested that Sesn2 and Nrf2 protein was involved in apoptotic death and dysfunction of EPCs induced by Ang-II while the underlying and potential mechanisms remained as unknown.

Sesn2 was reported to be able to protect human umbilical vein endothelial cells from apoptosis induced by extracellular Histones [49, 50]. Fang et al. have reported that patients with higher levels of angiotensin II (Ang II) and carotid atherosclerotic plaque (CAP), exhibited increased Sesn2 levels when compared with patients without these clinical characteristics [17]. However, the roles of Sesn2 in EPCs remained as to be illuminated. To investigate the role of Sesn2 in the apoptotic death of EPCs, Lenti-Sesn2 was designed to upregulate the levels of Sesn2. Upregulation of Sesn2 was able to repress apoptosis of EPCs induced by Ang-II treatments. Meanwhile, Sesn2 upregulation could also restore the impaired tube formation of EPCs induced by Ang-II. Recent research has suggested that there were links between Sesn2 and the Nrf2 pathway. In similarly related research, it was found that inducing forced expression of Sesn2 caused autophagic degradation of keap1 and promoted Nrf2 expression and activity [33]. In our experiments, we found that Keap1 was repressed by Ang-II and found that upregulation of Sesn2 further suppressed the levels of Keap1 proteins. We also found that the levels of Nrf2 proteins were increased by the treatments with Ang-II as well as found that upregulation of Sesn2 further enhanced the levels of Nrf2. The mechanisms we identified which influenced the dynamics Sesn2 and Nrf2 illustrated why Ang-II did not affect the levels of expression of Nrf2 mRNA. We postulated that Ang-II might have promoted the levels of Nrf2 proteins in EPCs as result of triggering processes meant to protect EPCs from Ang-II.

Nrf2 was reported to play an important role in stem cell state and function including redox homeostasis, metabolic reprogramming, survival, self-renewal, differentiation and so on [51]. For example, Nrf2 deficiency promotes hematopoietic stem cell (HSC) spontaneous apoptosis and reduces survival in response to oxidative stress [52]. Nrf2 overexpression increases mesenchymal stem cell (MSC) survival and resistance to oxidative stress [53, 54]. For EPCs, Nrf2 increases endothelial progenitor cell proliferation, survival and angiogenic function [51]. Nrf2 knockout can reduce the survival, proliferation, migration and angiogenesis potential of mouse pro-angiogenic cells, and affect the angiogenic transcriptome [55]. Silencing Nrf2 by siRNA can impair the biological functions of mouse EPC or human cord blood endothelial colony forming cells (ECFC), accelerate cell senescence, increase oxidative stress, and inhibit pro-angiogenic factors including VEGF, SDF-1 and NO [56–58]. The activation of Nrf2 improves the survival and angiogenesis of mouse EPC under diabetic conditions by increasing the nuclear translocation of Nrf2 mediated by CXCR7 expression and the up-regulation of its downstream antioxidant genes [59]. Many studies have reported that Sesn2 activated keap1/Nrf2 pathway to mediate various physiological and pathological process [31, 32, 60–62]. Therefore, Sesn2 may protect EPCs from Ang-II through Nrf2 pathway signal.

To confirm the link between Sesn2 and Nrf2, we designed si-Nrf2 with the ability such as to silence the expression of Nrf2. We found that silencing of Nrf2 was capable of reversing the protective effect of Sesn2 on EPCs. Importantly, we found that upregulation of Nrf2 was able to prevent EPCs from apoptosis and from dysfunction induced by Ang-II. These results indicated that Sesn2 protected EPCs from Ang-II through the Nrf2 signaling pathway. On the basis of these findings, we further investigated underlying mechanisms between Sesn2 and Nrf2. Autophagy was considered to have been an essential mediator between Sesn2 and Nrf2. In research similar to our own, there were likewise findings such that Sesn2 was found to have enhanced the levels of expression and activity of Nrf2 by way of promoting the P62-dependent autophagic degradation of Keap1 [33]. Our results demonstrated that restraint of autophagy could induce the reversal of the protective effects of Sesn2 on EPCs. Meanwhile, restraint of autophagy also weakened the relationship between Sesn2 and the Keap1/Nrf2 signaling pathway. We also found that by suppressing autophagy we caused a reduction in entry of Nrf2 into the nucleus induced by overexpression of Sesn2. Moreover, we found that suppression of autophagy further enhanced the role of si-Sesn2 in EPCs. These results revealed that autophagy acts as an intermediate molecule and Sesn2 protects EPCs via the Keap1/Nrf2 signaling pathway.

In own research and previous studies, Nrf2 was sound to have played a dramatic role as a transcription factor which promoted increased the expression of cytoprotective genes that acted against oxidative stress [63–66]. In the above-mentioned previous experiments, we found that Sesn2 was able to reduce the production of ROS induced by the treatments with Ang-II in EPCs. Our experiments demonstrated that the known ROS inhibitor, NAC, was able to protect EPCs from apoptosis and restore the impaired tube formation of EPCs when Nrf2 was silenced by si-Nrf2 in EPCs. These results revealed that Sesn2 promoted the survival and migration of EPCs exposed to Ang-II through the antioxidant functions of Nrf2. Interestingly, we also found that autophagy was increased by the treatments with si-Nrf2. We speculated that silencing of Nrf2 activated the fluxes in autophagy such as to promote the levels of expression of Nrf2. Moreover, we found that subsequently increased autophagy flux resulted in the elimination of damages to organelles caused by ROS in EPCs when Nrf2 was suppressed, which ultimately attenuated apoptosis and dysfunctions of EPCs. However, these hypotheses require further experiments to verify.

Results from Lao et al. [35] indicated that Sesn2 promoted HUVEC survival after the treatments with Ang-II. Our results also confirmed that Sesn2 protected EPCs form the treatments with Ang-II. Therefore, their results help to prove that our results were credible, and that Sesn2 protects endothelial cells from Ang- II. However, there are differences between their study and our own. First, their experiments were performed in HUVEC cell lines and our experiments were performed in primary endothelial progenitor cells. Second, Lao et al. [35] demonstrated that the JNK/c-Jun pathway acted as an upstream target to increase the levels of Sesn2 in HUVEC treated with Ang-II, whereas we focused upon the downstream aspects of Sesn2 in EPCs treated with Ang-II. Our results revealed that Sesn2 protected EPCs form Ang-II though effects upon the regulation of the Keap1/Nrf2 pathway. Thus, the novelty of our current work was reflected in the downstream mechanisms of Sesn2 in endothelial cells. There were some limitations in our study which limit some of the findings. Due to lack of funding, animal experiments in *vivo* were not performed. However, based on the methods we have outlined and the important findings we found, this would be a relatively easy avenue to complete follow-up research in future experiments. Based on our knowledge form the experiments we completed, we suggest that a good plan would be to construct a knocked-out version of Sesn2 in similar mice as we used for completion of such types of in vivo experiments.

**Figure 8:**
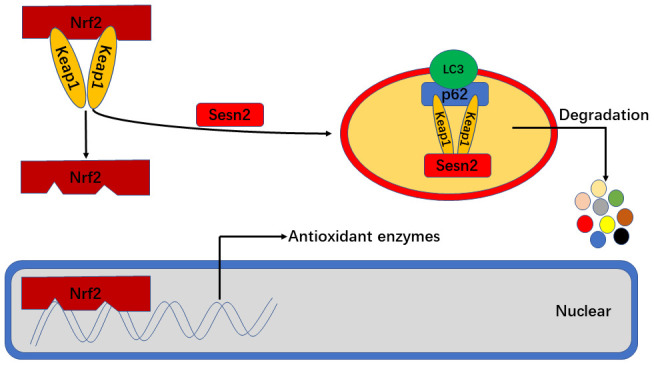
**Sesn2 serves as antioxidants by improving P62-dependent autophagic degradation of Keap1 and thereby promoting Nrf2 expression and activity.** When Sesn2 binds to P62 and Keap1, P62 binds to LC3 simultaneously at autophagosomes and thereby promote Keap1 degradation. The degradation of Keap1 was found to have led to Nrf2 expression and activation, and then promoted the transcription of genes for various antioxidant enzymes.

In conclusion, we found that Sesn2 improved the viability, tube formation ability, and migration of EPCs exposed to Ang-II by effecting changes in the dynamics of the Keap1/Nrf2 signaling pathway. It is known that Sesn2 activates the Keap1/Nrf2 signaling pathway by promoting the dynamics of P62-dependent autophagic flux. Further, our findings indicated that antioxidant functions of Nrf2 played an important role in resultant protective effects of Sesn2 on EPCs (Figure 8). One important finding in our research was the identification of novel underlying mechanisms related to Ang-II-induced apoptosis and dysfunction, as well as provided a potential therapeutic target for atherosclerosis.

## MATERIALS AND METHODS

### EPC isolation and culture from human umbilical cord blood

We isolated EPCs from samples of human umbilical cord derived blood by using density gradient centrifugation with Histopaque-1077 (Sigma). We gently added the samples of human blood to tubes pre-filled with separation solution and then centrifuged samples at 2000 rpm for 20 min at 4° C. After centrifugation, there were three resultant layers based upon differences in densities of constituents, including the uppermost layer which contained serum, the middle layer which contained white blood cells, and the lowermost layer which was contained red blood cells. During this process, the middle layer becomes suspended within the separation liquid. We removed white blood cells and constituents from the lowermost layer and individually resuspended them in complete endothelial cell growth medium (EGM)-2 (Lonza), followed by a final re-seeding of these samples into wells of six-well plates. We changed the medium every 72 hours, and we subcultured ECPs were according to 1:3 ratios cells to cells. We detected known biomarkers for EPCs by using immunofluorescence. For Immunofluorescence steps, we used primary antibodies against CD34, CD31, endothelial growth factor receptor 2 (VEGFR2), and CD133 which we purchased from Cell Signaling Technology. And then we detected the marker including CD34, CD133 and VEGFR2. The positive rate of CD34, CD133 and VEGFR2 was 75.9%±1.6, 48%±2.1 and 81.9%±1.9% respectively (Supplementary Figure 1). All aspects of our experiments were reviewed and approved by the Ethics Committee of Xinhua Hospital Affiliated to Shanghai Jiao Tong University School of Medicine. Further, all aspects of our experiments were performed according to and following all guidelines in the Helsinki Declaration. The IRB number for our study was XHEC-D-2018-368. Samples of blood derived from umbilical cord material was obtained from a single donor, and corresponding information attributed to the donor is shown in Table 1.

**Table 1:** The characteristics of donor.

**Characteristics**	**Patient**
Gender	female
Age	28 y, 8m and 9 d
Blood Pressure	117/82 mmHg
Glucose	5.4 mmol L^-1^
Heart Rate	77
Weeks of pregnancy	39
Special Medication	None
Tobacco	None
Alcohol	None

### Flow cytometry

Took newly isolated human umbilical cord blood mononuclear cells, added FITC-labeled anti-human CD34, PE-labeled anti- human VEGFR2 and APC-labeled anti-human 2 μL each, and sent the flow cytometer to the machine Detection.

### Western blotting

We extracted total proteins from ECPs by using RIPA (Radioimmunoprecipitation lysis buffer, Beyotime, China) containing PMSF (Phenylmethanesulfonyl fluoride, Beyotime, China). Concentrations of protein were measured by using the bicinchoninic acid (BCA) method (Beyotime, China) following all manufacturer protocols. Proteins were separated by using 10-15% SDS-PAGE. We electrophoretically transferred proteins to polyvinylidene difluoride (PVDF) membranes (Millipore, USA). Membranes were blocked with 5 % skim milk and were then incubated with primary antibodies for: Sesn2 (Proteintech, USA), Nrf2 (Proteintech, USA), Keap1 (Abcam, UK), Bax (Proteintech, USA), Bcl-2 (Proteintech, USA), Cytochrome c (Proteintech, USA), Cleaved-caspase 3 (Proteintech, USA), Cleaved-caspase9 (Abcam, UK), P62 (Proteintech, USA), LC-3 (Abcam, UK), Beclin-1 (Proteintech, USA), CD31 (Abcam, UK), CD34 (Abcam, UK), CD133 (Abcam, UK), VEGFR2 (Abcam, UK), Lamin B (Proteintech, USA) and GAPDH (Beyotime, China). We next incubated the membranes with secondary antibodies. Results for the membranes post-incubation periods were visualized by using chemiluminescence (Millipore).

### Real-time quantitative PCR

We extracted total RNA from EPCs by using TRIzol (TakaRa, China) according to all manufacturer protocols. For mRNA, we carried out reverse transcription and quantitative Real-Time polymerase chain reaction (qRT-PCR) as has been previously described. Briefly, we used extracted RNA for reverse transcription to produce cDNA by using a Prime-Script RT reagent kit with gDNA Eraser (Takara Biotechnology Co., Ltd., Japan) according to all manufacturer protocols. We carried out qRT-PCR out on a Roche LightCycler by using SYBR® Premix Dimer Eraser™ (Perfect Real Time; Takara, Japan) with coupled cDNA synthesis for the desired genes. The sequences for primer set we used are listed as follows: Sesn2 Forward: 5’-CTCATCACCAAGGAACACATC-3’, Reverse: 5’-CTCTGTTCACTAGGGGGTGTAG-3’; and Nrf2 Forward: 5’- ACGGTATGCAACAGGACATTGAGC-3’, Reverse: 5’ TTGGCTTCTGGACTT|GGAACCATG-3’.

### Cell viability assays

We used CCK-8 (Beyotime, China) to determine cell viability after treatments of Ang-II. EPCs (1 × 10^4^ cells/well) were suspended in 100 μL of fresh medium containing Ang-II and were then seeded. After applying the Ang-II treatments, we added CCK-8 (10 μL/well) the mix with freshened medium and incubated samples for 3 h. Next, we determined measures of the absorbance at 450 nm.

### Transwell assays

We used transwell assays in order to determine measures of the migration of EPCs. Complete fresh EGM-2 containing treatments of either Ang-II or CQ (each 600 μL) was added to the bottom chambers. We then added200 μL of serum-free medium containing 5 × 10^4^ EPCs to the top chambers. After incubating at 37° C for 12 h, EPCs transmigrating to membranes were fixed by using 4 % paraformaldehyde and were then stained with crystal violet. We selected three random microscopic fields to count stained cells.

### Wound healing assays

We used approximately 1 × 10^6^ EPCs per sample seeded into six-well plates and then incubated at 37° C. When EPCs reached a level of confluence of at least 90 %, we manually generated wounds by scratching cell monolayers with a sterile 200 μL plastic pipette tip and then incubated the samples in fresh medium containing 1 % FBS for 24 h. We took photographs to assess the mean distance of migration per field. The measured area of the remaining unhealed scratch wounds was calculated as follows: migration area (%) = (A0 – An)/A0 × 100, where A0 represents the area of the initial wound and an represents the remaining area of wound at the time step the corresponding measurement was made.

### Matrigel angiogenesis assays

Matrigel^TM^ (100 μL; BD Biosciences) was paved in the wells of 96-well plates and incubated at 37° C for 30 min. We then seeded 2 × 10^4^ EPCs on the Matrigel and incubated at 37° C for 8 h. Next, we photographed the results and produced images under magnification using microscopy (Leica, Germany), and analyzed the results by using Images J software.

### Immunofluorescence analysis

We seeded EPCs upon glass coverslips in a six-well plate and then fixed the samples by using 4 % paraformaldehyde diluted in PBS for 30 min. EPCs were permeabilized with 0.1% Triton-X-100 in PBS for 15 min, and subsequently blocked in 5 % goat serum for 1 h. We incubated EPCs in primary antibodies overnight at 4° C including at all of the following dilutions: CD31 (1:100, CST, USA), CD34 (1:200, CST, USA), CD133 (1:200, Abcam, UK), VEGFR2 (1:100, Abcam, UK), Keap1 (1:400, Proteintech, USA), Nrf2 (1:200, Proteintech, USA) and Caspase3 (1:200, CST, USA). We washed EPCs with PBST three times and then incubated the samples in Cy3-conjugated AffiniPure goat Goat anti-rabbit Anti-Rabbit IgG (H+L) and Alexa Fluor 488-conjugated AffiniPure goat anti-mouse Goat Anti-Mouse IgG (H+L). Measures of immunofluorescence were determined by using fluorescence microscopy (Olympus BX51).

### ROS measurement

We measured the levels of ROS production by using a Reactive Oxygen Species Assay Kit (Beyotime, China) following all manufacturer protocols. Briefly, we washed EPCs with PBS and incubated the samples with serum-free medium containing with DCFH-DA at 37° C for 20 min. Then the DCFH-DA in plates was carefully removed and discarded and we then washed EPCs with serum-free medium three times. We produced images for analysis using photography under microscopy (Olympus Fluoview, Japan) whereby positive cells were found to have emitted green color at various intensities.

### Hoechst 33258 staining

Apoptotic EPCs were identified by using Hoechst 33258 staining (Beyotime, China). We seeded EPCs at densities of 6 × 10^5^ cells/well in wells of a 60 mm plate. After applying the variously composed treatments, we fixed EPCs with 4 % paraformaldehyde for 10 min, then washed samples with PBS three times, followed by staining with 2 μg/mL Hoechst 33258 (Beyotime, China) in Hank’s balance salt solution for 5 min. Changes in the morphology of apoptotic nuclei were evaluated under fluorescence using microscopy (Olympus BX51) at an absorbance wavelength of 460 nm.

### Small interfering RNA transfection

Small interfering RNAs specifically targeting Sesn2 and Nrf2 were designed by and purchased from Gene Pharma, China. Si-Sesn2 and si-Nrf2 sequences are shown in Table 2. We mixed Si-Sesn2 and si-Nrf2 with Lipofectamine 2000 (Invitrogen, Carlsbad, California, USA) for 20 min in the serum-free medium. We then next added the mixtures to the plates.

**Table 2:** Sequences of small interfering RNAs.

**siRNA**	**Forward (5’-3’)**	**Reverse (5’-3’)**
Si-Sesn2-1	GCGAGAUCAACAAAUUACUTT	AGUAAUUUGUUGAUCUCGCTT
Si-Sesn2-2	CTCACACCATTAAGCATGGAG	CAAGCTCGGAATTAATGTGCC
Si-Sesn2-3	GCGAGAUCAACAAAUUACUTT	AGUAAUUUGUUGAUCUCGCTT
Si-Nrf2-1	CUAACUAUCUCGAACGCAACTCT	UUGCGUUCGAGAUAGUUAGCTCT
Si-Nrf2-2	AAGAGUAUGAGUGGAAAAACTT	GUUUUUCCAGCUCAUACUCUUTT
Si-Nrf2-3	CCCUGUGUAAAGCUUUCAATT	GAAAGCUUUACACAGGGTT

### Lentivirus transfection

The LV3-pGLV-H1+Puro plasmids with pcDNA-Sesn2 (Lenti-Sesn2), The LV3-pGLV-H1+Puro plasmids with pcDNA-Nrf2 (Lenti-Nrf2), and control Lentivirus (Lenti-NC) were all designed and synthesized by Gene Pharma, China. Briefly, we transfected ECPs with Lentivirus particle following all manufacturer protocols. The mRNA and protein levels of expression Sesn2 and Nrf2 were determined by using qRT-PCR and Western blotting.

### Statistical analyses

All data were presented as the mean ± standard deviation (SD). We used GraphPad Prism Software to analyze all data and statistical analyses. We used Student’s t-tests to analyze comparisons of the measures between two treatment groups. For multiple comparisons among multiple treatment groups, we used two-way analysis of variance (ANOVA) followed by Tukey’s post-hoc tests. The levels of statistical significance was at a P value of *p < 0.05, and values of **p < 0.01 and ***p < 0.001 were considered as increasingly statistically significant.

### Availability of data and materials

The datasets used and/or analyzed during the current study are available from the corresponding author upon request.

## Supplementary Material

Supplementary Figures
