# Supplementary material for: Sesn2 attenuates the damage of endothelial progenitor cells induced by angiotensin II through regulating the Keap1/Nrf2 signal pathway
Source: Aging (Albany NY). 2020 Nov 24;12(24):25505–27. doi: 10.18632/aging.104156 (PMC7803511; doi:10.18632/aging.104156)
Supplement: Supplementary Figures [file aging-12-104156-s001.pdf]

SUPPLEMENTARY FIGURES

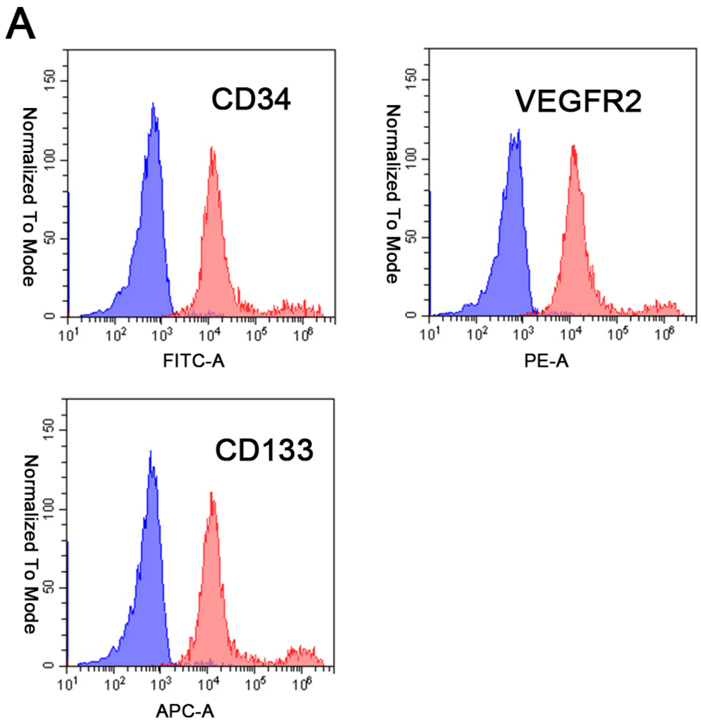

Supplementary Figure 1. (**A**) The flow cytometry was used to detect the marker of CD34, CD133 and VEGFR2. All experiments were performed in triplicate. *p < 0.05, **p < 0.01, ***p < 0.001, versus the control. Data are represented as mean +/- SEM.

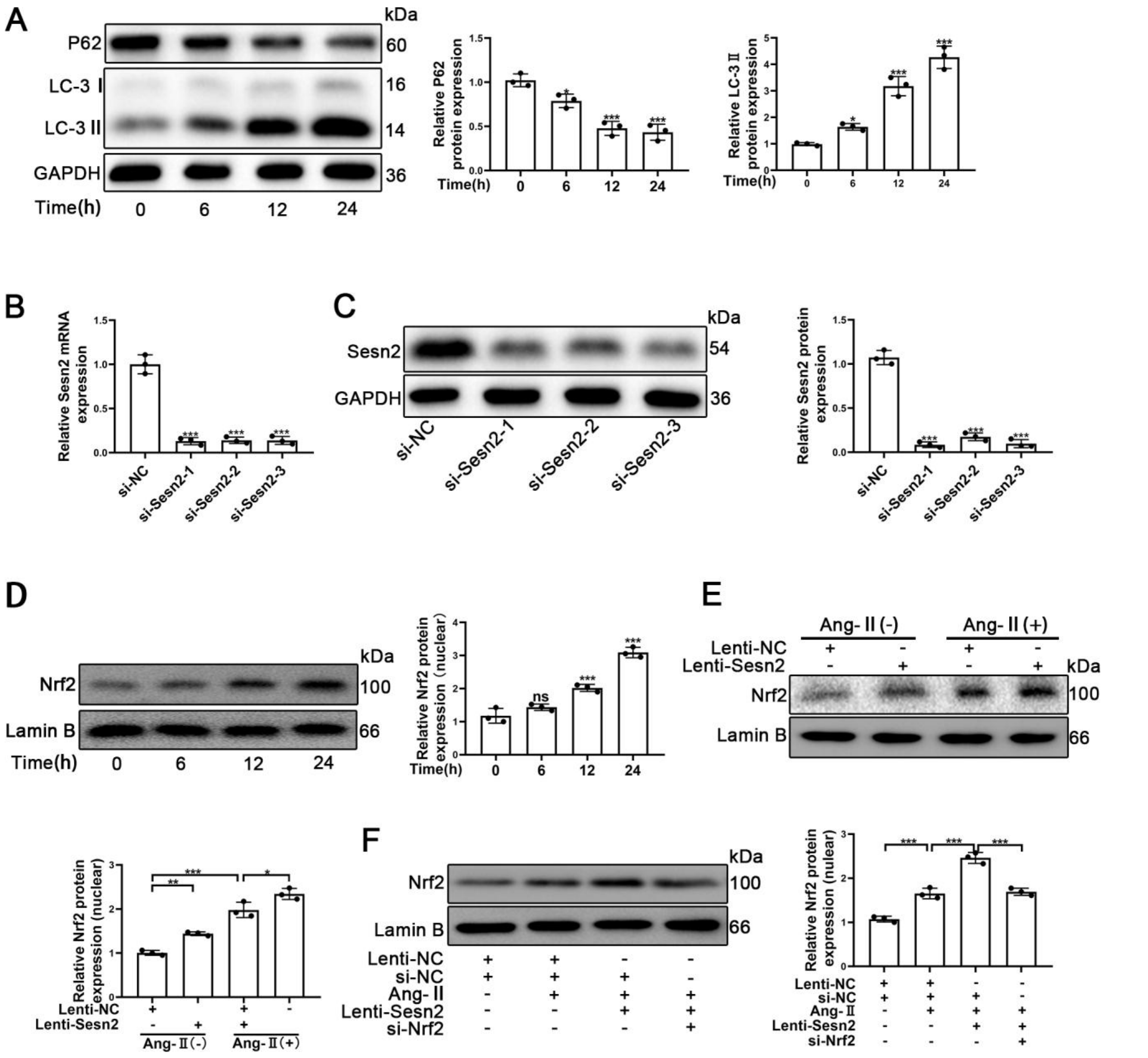

Supplementray Figure 2. (**A**) The application of Ang-II was found to have induced a decrease P62 protein expression and an increased LC-3II protein expression in EPCs. (**B**) The levels of Sesn2 mRNA were reduced by the treatments with si-Sesn2. (**C**) The levels of Sesn2 proteins were reduced by the treatments with si-Sesn2. (**D**) The levels of Nrf2 proteins were detected in the nuclear of EPCs exposed to Ang-II for prolonged periods of time. (**E**) The levels of Nrf2 proteins were detected in the nuclear of EPCs divided into Lenti-NC, Lenti-Sesn2, Lenti-NC + Ang-II and Lenti-Sesn2 + Ang-II treatment groups. (**F**) The levels of Nrf2 proteins were detected in the nuclear of EPCs divided into treatment groups including for a control, Ang-II, Ang-II + Lenti-Sesn2, and Ang-II+ Lenti-Sesn2 + si-Nrf2 treatment groups. All experiments were performed in triplicate. *p < 0.05, **p < 0.01, ***p < 0.001, versus the control. Data are represented as mean +/- SEM.

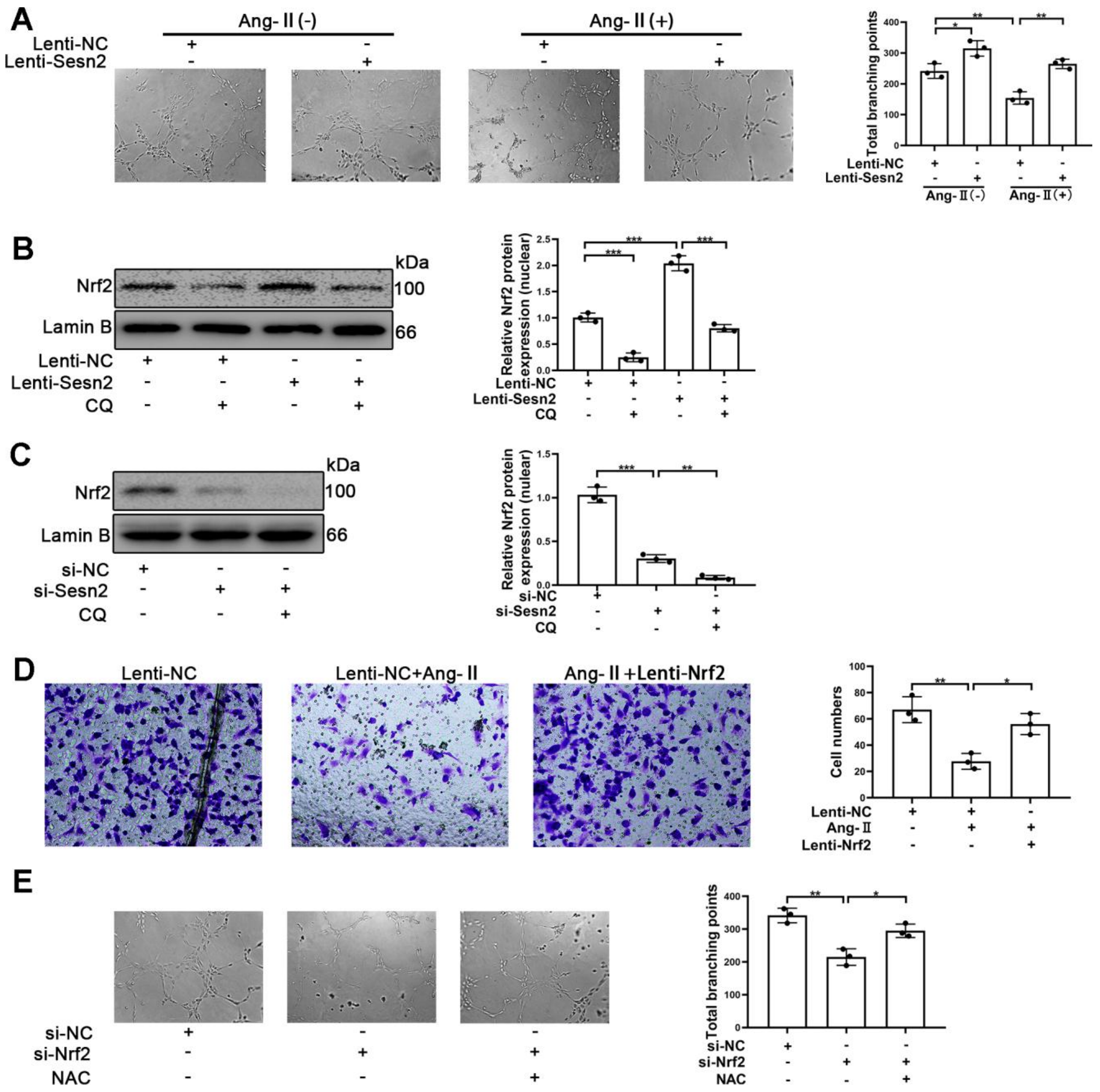

Supplementary Figure 3. (**A**) Tube formation abilities were assessed by Matrigel in EPCs detected in the nuclear of EPCs divided into Lenti-NC, Lenti-Sesn2, Lenti-NC + Ang-II and Lenti-Sesn2 + Ang-II treatment groups. Scale bars = 10 μm. (**B**) The levels of Nrf2 proteins were detected in the nuclear of EPCs divided into treatment groups representing the control, CQ, Lenti-Sesn2, and Lenti-Sesn2 + CQ cohorts. (**C**) The levels of Nrf2 proteins were detected in the nuclear of EPCs divided into treatment groups representing the control, si-Sesn2, and si-Sesn2 + CQ. (**D**) The migration of EPCs were determined by using Transwell assays in the control, si-Sesn2, and si-Sesn2 + CQ treatment groups. Scale bars = 20 μm. (**E**) Tube formation abilities were assessed by Matrigel in EPCs detected in the nuclear of EPCs divided into treatment groups representative of the control, si-Nrf2 and, si-Nrf2 + NAC. Scale bars = 10 μm. All experiments were performed in triplicate. *p < 0.05, **p < 0.01, ***p < 0.001, versus the control. Data are represented as mean +/- SEM.
